# Intravitreal injections as a leading cause of acute postoperative endophthalmitis—a regional survey in England

**DOI:** 10.1038/s41433-021-01886-3

**Published:** 2021-12-23

**Authors:** Ariel Yuhan Ong, Axelle Rigaudy, Shafak Toufeeq, Julian Robins, Zaid Shalchi, Mandeep Singh Bindra, Peter Charbel Issa

**Affiliations:** 1 Oxford Eye Hospital, Oxford University Hospitals NHS Foundation Trust, Oxford, UK; 2 Prince Charles Eye Unit, Royal Berkshire NHS Foundation Trust, Windsor, UK; 3 Royal Berkshire Hospital, Royal Berkshire NHS Foundation Trust, Reading, UK; 4 Milton Keynes University Hospital, Milton Keynes University Hospital NHS Foundation Trust, Milton Keynes, UK; 5 Stoke Mandeville Hospital, Buckinghamshire Healthcare NHS Trust, Aylesbury, UK; 6 Nuffield Laboratory of Ophthalmology, Nuffield Department of Clinical Neurosciences, University of Oxford, Oxford, UK

**Keywords:** Eye diseases, Medical research

## Abstract

**Background:**

To evaluate the characteristics, treatment patterns and outcomes of acute postoperative endophthalmitis.

**Methods:**

Patients presenting with acute postoperative endophthalmitis between January 2017 to December 2019 were identified from hospital records in this multicentre retrospective cohort study. Clinical records were reviewed for visual acuity (VA) at various timepoints, cause of endophthalmitis, microbiological results, treatments and complications.

**Results:**

Forty-six eyes of 46 patients were included. Intravitreal injections were the leading cause of acute postoperative endophthalmitis (*n* = 29; 63%), followed by cataract surgery (*n* = 8; 17%), vitreoretinal surgery (*n* = 7; 15%), and secondary intraocular lens insertion (*n *= 2, 4%). The absolute risk of endophthalmitis was 0.024% (1:4132) for intravitreal injections, 0.016% (1:6096) for cataract surgery, and 0.072% (1:1385) for vitreoretinal surgery. The majority of patients (*n* = 38; 83%) had better VA at 6 months compared to presentation, although fewer (*n* = 13; 28%) maintained similar or better VA compared to before the precipitating surgery. Twenty-four cases yielded positive culture results, of which *staphylococcus epidermidis* was the most commonly isolated organism. Microbiological yield was not associated with better final visual outcomes. Patients who underwent therapeutic vitrectomy (*n* = 15; 33%) had poorer VA at presentation, but subsequently achieved visual outcomes comparable to those who received medical treatment alone. There was no difference in time to presentation, visual outcome and retinal detachment rates among the different causative procedures.

**Conclusion:**

Intravitreal injections were the most common cause of endophthalmitis in our region, primarily because of their higher frequency compared to other intraocular procedures. In this cohort, the primary procedure had no effect on presentation, management or visual outcomes.

## Introduction

Endophthalmitis is an uncommon but devastating complication of intraocular procedures. Although the incidence of endophthalmitis is low [1–3] its potential precipitants are increasing in frequency year on year—cataract surgery is the most commonly performed elective surgical procedure in the United Kingdom (UK) today [4] and intravitreal injections are increasingly ubiquitous in treating a wide range of retinal conditions.

Controversies in the management of postoperative endophthalmitis exist. Fluid sampling from the vitreous cavity and intravitreal antibiotic injection (tap & inject) is an established first-line therapy [5]. The landmark Endophthalmitis Vitrectomy Study (EVS) in 1995 concluded vitrectomy may be beneficial in post-cataract surgery endophthalmitis presenting with light perception vision or worse. However, there is currently no consensus regarding the role of early vitrectomy using advanced surgical techniques in patients with better vision, the diagnostic value of anterior chamber taps, the clinical utility of systemic antibiotics, systemic versus local (periocular, intravitreal) steroids, and if management should differ depending on the aetiology of the endophthalmitis [6–8].  The comparability of studies on acute postoperative endophthalmitis is affected by the heterogeneity of diagnostic criteria, microbiological results, and treatment protocols, which may affect the generalisability of the results to our daily clinical practice [9–11].

Herein, we evaluate the contemporary presentation and management of acute postoperative endophthalmitis from all causes in a relatively homogenous, real-world, National Health Service (NHS) setting. We study the procedure-related absolute risk, clinical presentation, practice patterns, microbiological results and treatment outcomes across the Thames Valley region in the UK over a 3-year period.

## Methods

This was a multicentre retrospective study involving seven hospitals across all four hospital trusts in the Thames Valley region, comprising Oxford University Hospitals NHS Foundation Trust, Buckinghamshire Healthcare NHS Trust, Milton Keynes University Hospital NHS Foundation Trust, and Royal Berkshire NHS Foundation Trust. This was undertaken under the auspices of the Oxford Trainee Research Network, a collaborative network of ophthalmology trainees across the Thames Valley Deanery. The study was registered as a clinical audit with all the hospital trusts involved and was exempt from requiring formal ethics approval.

### Patient selection and management

All consecutive patients presenting with acute postoperative endophthalmitis from 1 January 2017 to 31 December 2019 were identified. This was defined as severe intraocular inflammation due to infection occurring within 6 weeks of any surgery or invasive ocular procedure. The initial diagnosis was made clinically, based on signs and symptoms associated with endophthalmitis such as pain, redness, loss of vision, hypopyon, intraocular inflammation, hazy media [5] supported by adjunctive B-scan ultrasonography. All patients were reviewed by senior ophthalmologists prior to initiating treatment. To ensure completeness and accuracy of case ascertainment, several methods were used and cross-referenced against each other: local endophthalmitis logbooks, local critical incident reporting logs and diagnoses recorded in the departmental electronic medical record systems.

For the purposes of the study, cases with a negative microbiological yield or a disputed diagnosis were reviewed for the possibility of sterile inflammation such as toxic anterior segment syndrome (TASS) or non-infectious uveitis, and were subsequently excluded from analysis if clinical presentation did not meet the diagnostic criteria defined above. Patients with late/chronic endophthalmitis (over 6 weeks), endogenous endophthalmitis, as well as cases precipitated by trauma were outside the remit of this study and were also excluded.

### Data extraction and outcomes

Pseudo-anonymised data were extracted from the local departmental electronic medical record systems or paper records. This included demographic data (age at presentation, gender, comorbidities); clinical characteristics (aetiology, presenting symptoms, investigations, treatment, microbiological results); and visual acuity (VA) at baseline (last recorded prior to the intervention that caused endophthalmitis), presentation and follow-up. The follow-up schedule was determined by the treating team. We selected 6 months as the final follow-up time point.

Logarithm of the minimum angle of resolution (LogMAR) VA was used to facilitate analysis. In cases where VA was recorded in Snellen fractions, this was converted to the appropriate LogMAR equivalent. In accordance with the convention utilised by the National Ophthalmology Database audit, counting fingers (CF), hand movements (HM), perception of light (PL) and no perception of light (NPL) were assigned LogMAR equivalents of 2.1, 2.4, 2.7, and 3.0, respectively [4].

### Statistical analysis

All data were collected and recorded in a standardised Microsoft Excel spreadsheet (Microsoft Corporation, Redmond, WA). Data analysis was conducted using IBM SPSS Statistics for Mac, version 26 (IBM Corporation, Armonk, NY, USA). Continuous data were described with the median and interquartile range (IQR). The *χ*^2^ test was employed to conduct univariate analyses for categorical data, and the Mann-Whitney *U* test, Kruskal-Wallis test or Wilcoxon signed ranks test for non-parametric data as appropriate. The Shapiro-Wilk test confirmed a non-normal distribution. Multivariate analyses were performed to further evaluate significant outcomes. Binary logistic regression was employed to calculate adjusted odds ratios (OR) and 95% confidence intervals (CI) for binary outcomes, and the final model was assessed for fit using the area under receiver operating characteristic curve (AUROC). Outcomes were considered statistically significant at *p* < 0.05.

## Results

We identified 53 eyes of 53 patients with the recorded diagnosis of acute postoperative endophthalmitis. A detailed review of these cases led to exclusion of 7 eyes (4 cases of TASS, 2 sterile endophthalmitis related to intravitreal triamcinolone injections, 1 panuveitis), resulting in 46 eyes eligible for analysis.

**Fig. 1:**
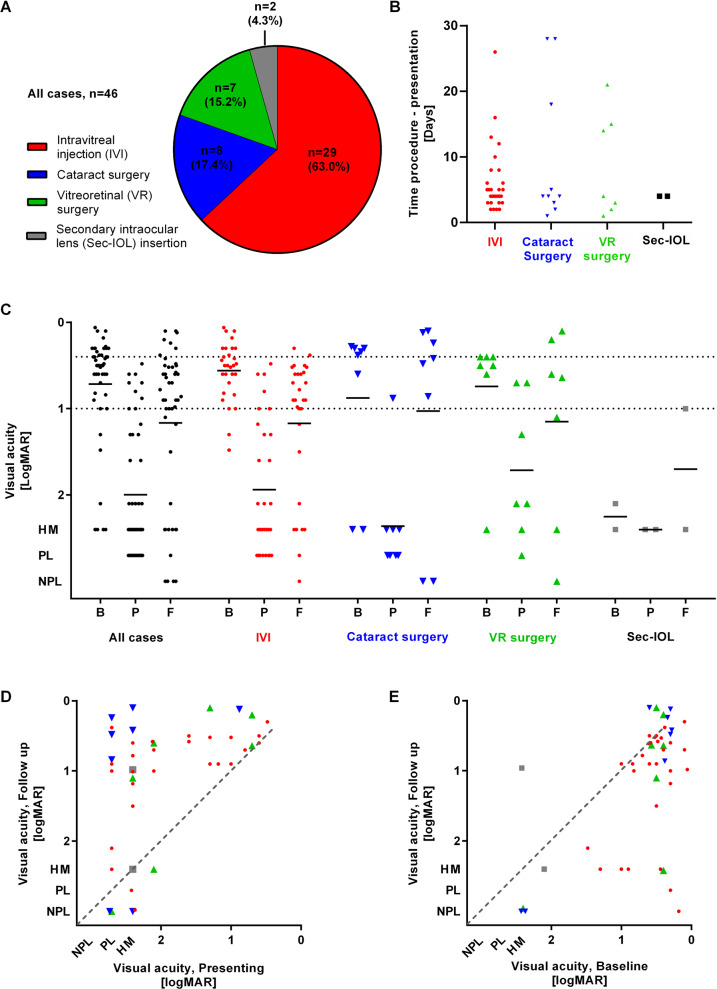
Graphical representation of causes, time to presentation, and visual acuity data for endophthalmitis cases. **A** Distribution of endophthalmitis cases by primary causative procedure. **B** Distribution of time from intraocular procedure to presentation, classified by primary causative procedure. **C** Distribution of visual acuity across all cases, then separately by primary causative procedure. **D** Scatter plot of visual acuity at baseline compared to follow-up at 6 months. **E** Scatter plot of visual acuity at presentation compared to follow-up at 6 months. B, Baseline; P, Presenting; F, Final.

The 46 patients had a median age of 76.6 years (IQR 69.8–86.9) with a fairly even gender distribution (25/46 females, 54%). Intravitreal injections accounted for the majority of cases (29/46, 63%), followed by cataract surgery (8/46, 17%), and vitreoretinal surgery (7/46, 15%; no combined intraocular procedures) (Fig. 1A). Secondary intraocular lens (IOL) insertion comprised a small minority (2/46, 4%). No cases of endophthalmitis related to glaucoma or corneal surgeries presented within 6 weeks of the surgery. The absolute risks for the different interventions in this sample are provided in Table 1.

**Table 1:** Incidence of endophthalmitis for the 3-year period spanning 2017–2019, classified by the three most common primary causative procedures.

	Total Procedures	Cases of Endophthalmitis		Absolute Risks (Incidence)	
		Total	per 10,000		
Cataract surgery	42,671	7	1.64	0.016%	1: 6,096
Intravitreal injections	115,682	28	2.42	0.024%	1: 4,132
Vitreoretinal surgery	6,926	5	7.22	0.072%	1: 1,385

The numbers of endophthalmitis cases per procedure differ from Fig. 1A because four cases resulting from procedures performed at external hospitals were excluded.

Of the patients with injection-related endophthalmitis, 15/29 were being treated for neovascular age-related macular degeneration (nAMD), 8/29 for diabetic macular oedema, 5/29 for macular oedema secondary to a retinal vein occlusion, and 1 for myopic choroidal neovascular membrane. With regard to vitrectomy-related endophthalmitis, the indications for the original procedure were epiretinal membrane (4/7) and 1 each of vitreous haemorrhage, macular hole, and diabetic tractional retinal detachment. The majority (6/7) were 25-gauge vitrectomies, 6/7 were self-sealing, and only 2 cases required tamponade agents (C3F8 and silicon oil).

The median time from intraocular procedure to presentation was 4 days (IQR 3–8.5), and was comparable across the various procedures (*p* = 0.97). Thirty-three patients (72%) presented within 7 days and 39 (85%) within 14 days of the procedure (Fig. 1B).

The median time from symptom onset to presentation was 2 days (IQR 1–3), with only 1 outlier presenting more than 1 week after symptom onset (19 days). There was no difference across the different precipitating procedures (*p* = 0.53).

### Treatment practice

A ‘tap and inject’ procedure (sampling of intraocular fluids followed by intravitreal antibiotic injection) was performed in 41 patients on the day of presentation. Of these, 11 (24%) had both anterior chamber (AC) and vitreous taps, 29 (63%) had vitreous taps alone, and 1 (2%) had AC tap alone. Two patients had intravitreal antibiotic injections without fluid sampling. Three patients proceeded straight to 3-port pars plana vitrectomy (PPV) with vitreous biopsy. Overall, 15 patients (33%) underwent at least one vitrectomy, of which 9 (60%) were considered early (within 7 days of presentation). The median time to vitrectomy was 4 days (IQR 1–15).

All 46 patients received standardized doses of intravitreal ceftazidime and vancomycin, or amikacin and vancomycin in cases of penicillin allergy (one patient only). Seven patients (15%) had a repeat course of intravitreal antibiotics within 48 h due to lack of treatment response. Oral antibiotics (ciprofloxacin or moxifloxacin) were used in the majority of cases (37/46, 80%), with a third of patients also receiving oral corticosteroid therapy (18/46, 39%) according to local protocols. Most patients were admitted to hospital for further management (40/46, 87%).

### Visual acuity

All patients had a minimum of 6 months of follow-up. Visual acuity data are illustrated in Fig. 1C. At presentation with acute endophthalmitis, VA was better than CF in 14 patients (30%), CF in 6 (13%), HM in 13 (28%) and PL in 13 (28%). No patients presented with NPL vision. At 6 months, 11 (24%) eyes achieved a good level of functional vision (final VA of LogMAR 0.5 or better), while 14 (30%) eyes were deemed to have achieved a poor visual outcome (‘severe vision impairment’ or LogMAR 1.0 or worse, as defined by the World Health Organisation) [12]. There was no statistically significant difference in presenting VA (*p* = 0.23) or final VA (*p* = 0.35) across the different primary causative procedures.

The majority of patients (38/46, 83%) had better VA at 6 months compared to presentation with acute endophthalmitis, with 33 (72%) experiencing an improvement of 2 or more lines of vision (Fig. 1D). Compared to their baseline VA (last recorded VA prior to procedure leading to endophthalmitis), 24 patients (52%) lost 2 or more lines of vision by 6 months (Fig. 1E).

Logistic regression analysis was conducted to examine the associations between a poor visual outcome (LogMAR ≥ 1.0) and various potential explanatory variables. Primary causative procedure, therapeutic vitrectomy, treatment with oral steroids or oral antibiotics, age and gender did not demonstrate statistical significance. Only a presenting VA of PL was associated with a poor visual outcome, which held true after adjusting for relevant covariates (adjusted model: OR 4.33, 95% CI 1.1–17.2, *p* = 0.04 AUROC = 0.88). Time from symptom onset to treatment was not included in this analysis because this was heavily skewed towards early presentation, precluding meaningful patient stratification.

**Fig. 2:**
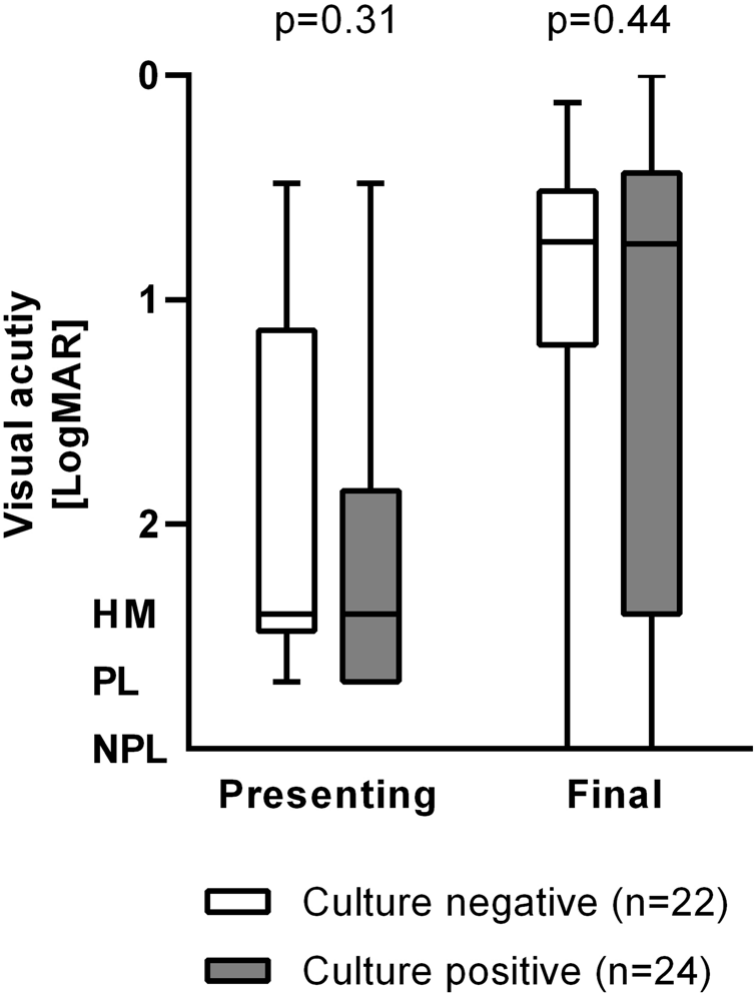
Visual acuity measurements by microbiological yield, at presentation and at final follow-up.

Patients who underwent therapeutic vitrectomy tended to have poorer VA at presentation (*p* = 0.04). These patients subsequently achieved visual outcomes comparable to those who received medical treatment alone (*p* = 0.67) (Fig. 2). There was an even distribution of patients undergoing vitrectomy across all causative procedures (*p* = 0.70).

### Microbiology

**Fig. 3:**
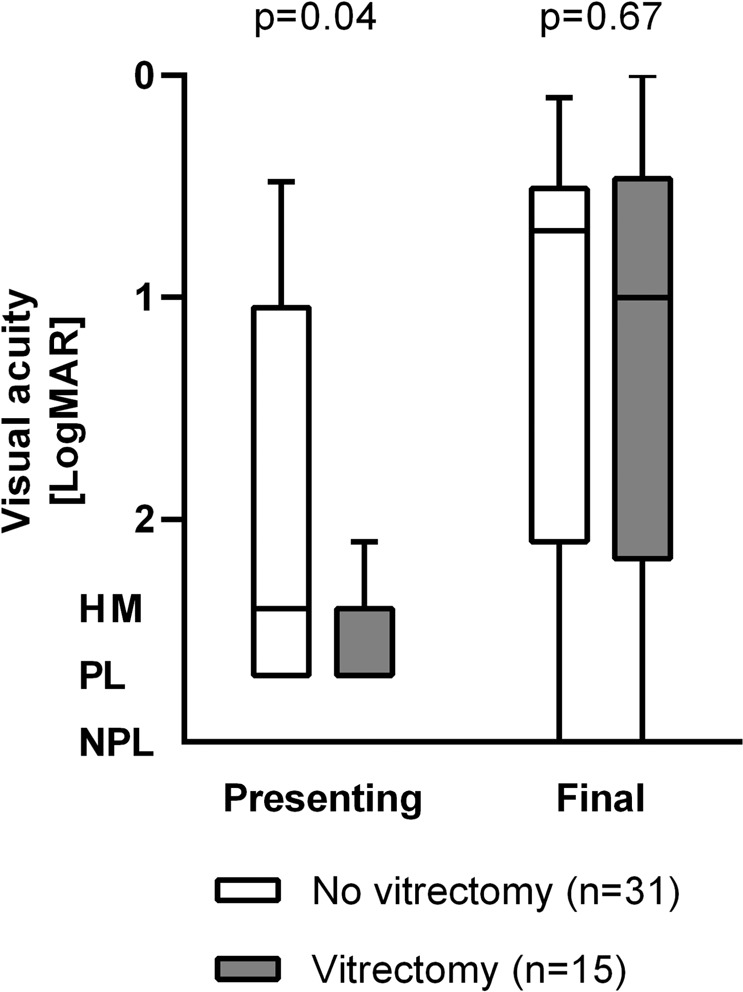
Visual acuity measurements by therapeutic vitrectomy, at presentation and at final follow-up.

Microbiology test results were available for 44 cases. Of these, 24 (55%) cases yielded positive culture results. AC taps did not yield any additional information beyond that provided by vitreous samples (AC and vitreous tap performed in 11 patients; AC tap was positive in only 4/6 culture-positive cases). All cases were bacterial, with no cases of fungal or viral endophthalmitis observed within the study period. The majority were gram-positive organisms (20/24, 83%). *Staphyloccocus epidermidis* was the most commonly isolated organism (10/24, 42%), followed by *staphylococcus aureus* (4/24, 17%) (Table 2). There was no significant difference in presenting VA or final VA based on microbiological yield (*p* = 0.31 and *p* = 0.44) (Fig. 3). In the subset of patients with culture-positive endophthalmitis, all cases with gram-negative bacteria had a final VA of LogMAR 1.0 or worse, and those that grew gram-positive bacteria tended to have better visual outcomes (*p* = 0.03).

**Table 2:** Microbiological results for patients with positive cultures.

Bacteria	*N* (%)
Polymicrobial (All gram positive)	3 (12.5)
*Streptococcus oralis, S. salivarius*	1
*Streptococcus oralis, S. sanguis*	1
*Corynebacterium striatum, C. amycolatum*	1
Gram Positive	17 (70.8)
*Staphylococcus epidermidis*	10
*Staphylococcus aureus*	4
*Staphylococcus capitis*	1
*Staphylococcus hominis*	1
*Streptococcus cristatus*	1
Gram Negative	4 (16.7%)
*Haemophilus influenzae*	1
*Acinetobacter Lwoffii*	1
*Serratia marcescens*	1
*Pseudomonas aeruginosa*	1

### Complications and Outcomes

In terms of complications, 1 eye (2%) became phthisical, 1 (2%) required an evisceration (the patient who presented 19 days after symptom onset) and 7 (15%) subsequently developed retinal detachments over at least 6 months of follow-up. On univariate analysis, there was no association between developing a retinal detachment and the primary causative procedure (*p* = 0.43). Retinal detachment occurred more frequently in patients who had a therapeutic vitrectomy, although this was not statistically significant (4/15 vs 3/31, *p* = 0.13).

Approximately half of the patients (15/29; 48%) with intravitreal injection-related endophthalmitis subsequently resumed treatment. Treatment cessation in the remainder were due to poor prognosis (9/29, 31%), disease inactivity (nAMD) (3/29, 11%) or patient refusal (2/29, 7%).

## Discussion

In this regional survey of all-cause acute postoperative endophthalmitis in the UK, intravitreal injections were the most common cause, comprising just over half of the cases and far exceeding that due to cataract surgery. Most previous studies examining endophthalmitis from different procedures reported cataract surgery as the leading cause of postoperative endophthalmitis [10, 13, 14] although a recent study from Sweden also found a higher proportion of cases caused by intravitreal injections [15]. The absolute risks of post-cataract and post-injection endophthalmitis were comparable in our study, meaning that the difference is due to the approximately three times higher number of intravitreal injections compared to cataract surgery performed in the region. Previous studies have demonstrated a rapid increase in the use of intravitreal injections in England [16], and the demographic changes and increasing number of licensed indications suggests the need for intravitreal injections will continue to grow. Hence, intravitreal injections may now be the leading cause of acute postoperative endophthalmitis.

In our patient cohort, the incidence of endophthalmitis related to injections was within the expected range [3, 17], but the incidence related to cataract surgery (0.016%) was rather low compared to many previous studies (0.024–0.265%) [2, 18–20]. This may reflect recent improvements in surgical technique and prophylaxis protocols. For example, the evidence for perioperative use of povidone–iodine [21], or the seminal ESCRS study [18] which introduced intracameral cefuroxime prophylaxis for cataract surgery over a decade ago, are both now de rigueur in clinical practice across all hospital sites included in the study (with or without postoperative topical antibiotic therapy).

No cases of acute postoperative endophthalmitis resulting from corneal or glaucoma surgery were identified in this study. This may be related to lower case volumes or later presentations (most cases of endophthalmitis secondary to corneal surgery develop after 6 weeks [22] and can present several years after glaucoma drainage implant or filtration surgery [23]) meaning that they were not within the remit of the study. There was insufficient data to calculate the true incidence of endophthalmitis resulting from secondary IOL insertion, although this umbrella term covers several heterogenous procedures which may make any result difficult to interpret.

While the risk of acute postoperative endophthalmitis remained very low across all types of intraocular procedures, we found the risk from vitreoretinal surgeries to be three times higher overall. The incidence of PPV-related endophthalmitis has ranged from 0.021 to 0.142% in the literature [24], although few studies have compared the risks across different procedures contemporaneously in the same institutional setting. Malmin et al. [15]. similarly found higher rates of PPV-related endophthalmitis over a 20-year study period (compared to cataract surgery and intravitreal injections). Some authors have speculated that unsutured sclerotomy wounds in small-gauge vitrectomy, which is the standard technique in all our study centres, may confer a higher risk of endophthalmitis [25], but this has been disputed by others [24, 26]. Unlike the ESCRS study which established best practice for antibiotic prophylaxis in cataract surgery, there has not been an equivalent study in vitreoretinal surgery [18]. Given the small numbers included in this study, it is important not to overstate the significance of our finding at this time, before similar data is replicated in other cohorts.

Whilst the EVS has advanced our understanding of the initial management of post-cataract surgery endophthalmitis, over two decades later, it remains the only randomised controlled trial (RCT) on the subject [27]. The EVS found primary vitrectomy in eyes presenting with PL vision tended to result in better visual outcomes compared to the ‘tap and inject’ group, with 56% versus 30% achieving VA of 20/100 or better. Amongst our cohort of patients with various procedures preceding endophthalmitis, those selected for therapeutic vitrectomy tended to have a poorer VA, but subsequently, their final VA was comparable to those who did not undergo vitrectomy. Although the numbers are too small to draw any definitive conclusions, this suggests a potential benefit to therapeutic vitrectomy in terms of VA gains. Other recent real-world studies have come to similar conclusions [14, 28], although the findings should be interpreted with caution as there was no control group (medical management alone) to compare with. Notably, in our cohort, over a quarter (4/15) of those who underwent a vitrectomy subsequently developed a retinal detachment, similar to one of the aforementioned studies (24%) [28], whereas the other found lower rates of intra- and post-operative retinal detachments (9%) [14]. The decision to proceed with vitrectomy should therefore be carefully balanced against a significant risk of complications, especially as the most appropriate visual threshold and/or patient cohort remains unclear. An RCT evaluating its role in managing postoperative endophthalmitis has been proposed and may help shed further light on this [29].

The microbiological yield of 55% in our study is in line with previous literature (yield range 18–64%) [30–32], with coagulase-negative *Staphylococci* as the commonest causative organisms. The relatively low rates of *Streptococcal* infections (which has been associated with poorer visual outcomes [30, 33]) in our study might be explained by the standardised use of surgical face masks to reduce droplet transmission from oral contaminants [34], although this remains a point of contention [35].

Varying definitions of postoperative endophthalmitis need to be considered when interpreting results, especially due to mimics such as TASS or sterile endophthalmitis [36, 37]. These cases are often initially treated as infectious endophthalmitis, although the clinical picture and better prognosis can help differentiate these entities [38]. Interestingly, the eligibility criteria used to define endophthalmitis was fairly variable in the literature. This ranged from the precision of the ESCRS study, which reviewed all culture-negative cases to exclude cases of TASS, to studies where the diagnosis was made on clinical grounds alone [13, 14, 30], to one which defined endophthalmitis as excessive postoperative inflammation regardless of treatment response or culture results [39]. This underscores the importance of careful case definition in determining treatment outcomes. We therefore sought to emulate the rigour of the ESCRS study by evaluating for the possibility of non-infectious intraocular inflammation and excluding them from the analysis [18].

Another strength of this study was its multicentre nature, which encompassed four large NHS trusts in the UK with fairly homogenous treatment practices. With the benefit of fairly complete outcome data, this represents the largest regional survey of acute postoperative endophthalmitis due to all causes in the UK, and provides insights into the distribution and case mix seen in clinical practice. The collaborative approach facilitated by our trainee research network adds to the growing body of evidence this is a feasible model for collecting and analysing data on rare conditions over multiple sites [19]. Limitations include the retrospective study design and the low number of included cases, which was necessitated by the relative rarity of the condition, and illustrates the need for further larger multicentre prospective studies with rigorous case definitions.

### Summary

#### What was known before

- Acute postoperative endophthalmitis is an uncommon postoperative complication with devastating implications. Although the risk has decreased with improved prevention, cases are likely to increase due to the increasing number of intraocular interventions.
- Acute postoperative endophthalmitis is often studied in isolation by cause, with heterogeneity of diagnostic criteria and management between studies.
- Direct comparison of incidence, frequency, characteristics, and outcomes across various different causative procedures in the same healthcare setting is scarce.

#### What this study adds

- The absolute risk of endophthalmitis was similar for cataract surgery (0.016%) and intravitreal injections (0.024%), but higher for vitrectomies (0.072%).
- Intravitreal injections are now the most common cause of endophthalmitis due to the high number of procedures.
- In this cohort, the primary causative procedure had no effect on presentation, management or visual outcomes.
- The study exemplifies that a collaborative trainee-led approach is a feasible model for collecting and analysing data on uncommon conditions over multiple sites.
